# Exercise reduces metabolic burden while altering the immune system in aged mice

**DOI:** 10.18632/aging.202312

**Published:** 2021-01-06

**Authors:** Kyeong Jin Yoon, Aram Ahn, Soo Hong Park, Seung Hee Kwak, Seong Eun Kwak, Wonsang Lee, Yong Ryoul Yang, Minji Kim, Hyun Mu Shin, Hang-Rae Kim, Hyo Youl Moon

**Affiliations:** 1 Department of Physical Education, Seoul National University, Gwanak-ro, Gwanak-gu, Seoul, Republic of Korea; 2 Institute of Sport Science, Seoul National University, Gwanak-ro, Gwanak-gu, Seoul, Republic of Korea; 3 Department of Kinesiology, University of Connecticut, Storrs, CT 06269,USA; 4 School of Kinesiology, University of Michigan, Ann Arbor, MI 48109, USA; 5 Aging Research Center, Korea Research Institute of Bioscience and Biotechnology, Daejeon, Republic of Korea; 6 Department of Biomedical Sciences, Seoul National University College of Medicine, Seoul, Republic of Korea; 7 BK21Plus Biomedical Science Project, Seoul National University College of Medicine, Seoul, Republic of Korea; 8 Medical Research Institute, Seoul National University College of Medicine, Seoul, Republic of Korea; 9 Wide River Institute of Immunology, Seoul National University, Hongcheon, Republic of Korea; 10 Institute on Aging, Seoul National University, Gwanak-ro, Gwanak-gu, Seoul, Republic of Korea

**Keywords:** exercise, immunosenescence, NK cell, aging, metabolism

## Abstract

Although several evidence has suggested the impact of exercise on the prevention of aging phenotypes, few studies have been conducted on the mechanism by which exercise alters the immune-cell profile, thereby improving metabolism in senile obesity. In this study, we confirmed that 4-week treadmill exercise sufficiently improved metabolic function, including increased lean mass and decreased fat mass, in 88-week-old mice. The expression level of the senescence marker p16 in the white adipose tissue (WAT) was decreased after 4-weeks of exercise. Exercise induced changes in the profiles of immune-cell subsets, including natural killer (NK) cells, central memory CD8^+^ T cells, eosinophils, and neutrophils, in the stromal vascular fraction of WAT. In addition, it has been shown through transcriptome analysis of WAT that exercise can activate pathways involved in the interaction between WAT and immune cells, in particular NK cells, in aged mice. These results suggest that exercise has a profound effect on changes in immune-cell distribution and senescent-cell scavenging in WAT of aged mice, eventually affecting overall energy metabolism toward a more youthful state.

## INTRODUCTION

Aging provokes diverse physiological changes in the functional and structural aspects of the body including decreased muscle size, bone density, and increased visceral fat [1, 2]. The risks of cardiovascular disease, type 2 diabetes, and cancer increase with age, along with vulnerability to viral infections, and the probability of vaccine failure, which increases overall mortality [3]. Seven pillars have been used to describe the mechanisms of aging: inflammation, stem cell regeneration, metabolism, proteostasis, macromolecular damage, adaptation to stress, and epigenetics [4]. However, the causes and underlying mechanisms of aging and rejuvenation have yet to be fully elucidated. In particular, little is known about metabolic rejuvenation in aging.

Metabolic function appears to be impaired by aging [5]; the reduced aerobic capacity and increased glucose intolerance that occur with aging impair metabolic function [6]. Adipose tissue plays an important role in energy storage and helps maintain homeostasis in the endocrine and immune systems via secreting multiple hormones and adipokines [7]. However, adipose tissue undergoes many alterations with aging, including accumulation of senescent cells, infiltration of immune cells, and increased secretion of pro-inflammatory cytokines and chemokines induced by aging, which causes systemic metabolic dysfunction [7, 8]. Impairment of adipose tissue increases the possibility of metabolic diseases such as type 2 diabetes and cardiovascular diseases [9].

Aging induces various changes in the body with respect to immunity along with these metabolic disorders [3]. Age-associated alterations in the immune system are generally referred to as immunosenescence, which includes impaired T-cell responses, modified B-cell subsets, and diminished natural killer (NK) cell and macrophage activities [10–15]. The proliferation and cytotoxicity of NK cells decrease with aging, which results in reduced recognition and elimination of senescent cells [16]. Macrophages in adipose tissue and the stromal vascular fraction (SVF) are associated with a chronic low-level inflammatory state in the elderly and appear to be highly related to immune and metabolic function, expressing elevated levels of pro-inflammatory cytokines, such as tumor necrosis factor α, interleukin (IL)-1, and IL-6 [17–23]. In particular, M1 macrophages in adipose tissue have been reported to increase the secretion of pro-inflammatory cytokines, which cause insulin resistance by disrupting insulin signaling and glucose uptake [8, 24, 25].

Exercise and physical activity have been reported to prevent and reverse aging-like phenotypes [26–28]. In particular, exercise improves cardiovascular function, muscle strength, and posture stability and reduces metabolic disease risk factors [29, 30]. Aerobic exercise directly affects the distribution and function of fat, causing a positive change in body composition by reducing total and visceral fat mass, and contributes to reduced secretion of inflammatory cytokines [31]. Exercise also affects the immune system [32]. Exercise increases the secretion of anti-inflammatory cytokines, such as IL-2, IL-4, and IL-10 [10]. In addition, chronic exercise training inhibits macrophage infiltration into adipose tissue and induces an acceleration of phenotypic switching from M1 to M2 macrophages [33]. However, several studies have assessed how exercise changes the immunity and inflammation-related content of adipose tissue and SVF in aging and how it relates to metabolism. We therefore evaluated alteration in the immune system and metabolic phenotypes of aged mice subjected to exercise.

## RESULTS

### Effect of exercise on metabolic phenotypes of aged mice

**Figure 1:**
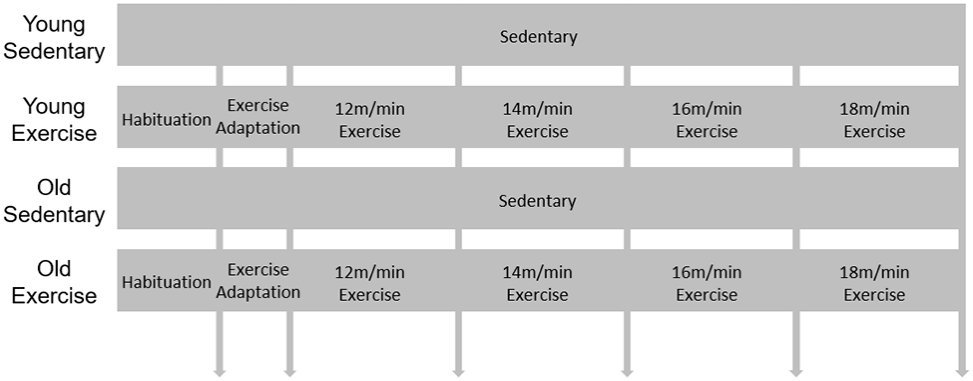
**Schematic design for exercise protocol.** 9-week-old and 84-week-old mice were divided into sedentary or exercise group. After 1day habituation in the room where treadmill is, mice were adapted in treadmill for 2 days. Then speed of treadmill was gradually increased (2 m/min weekly) during 4 weeks.

**Figure 2:**
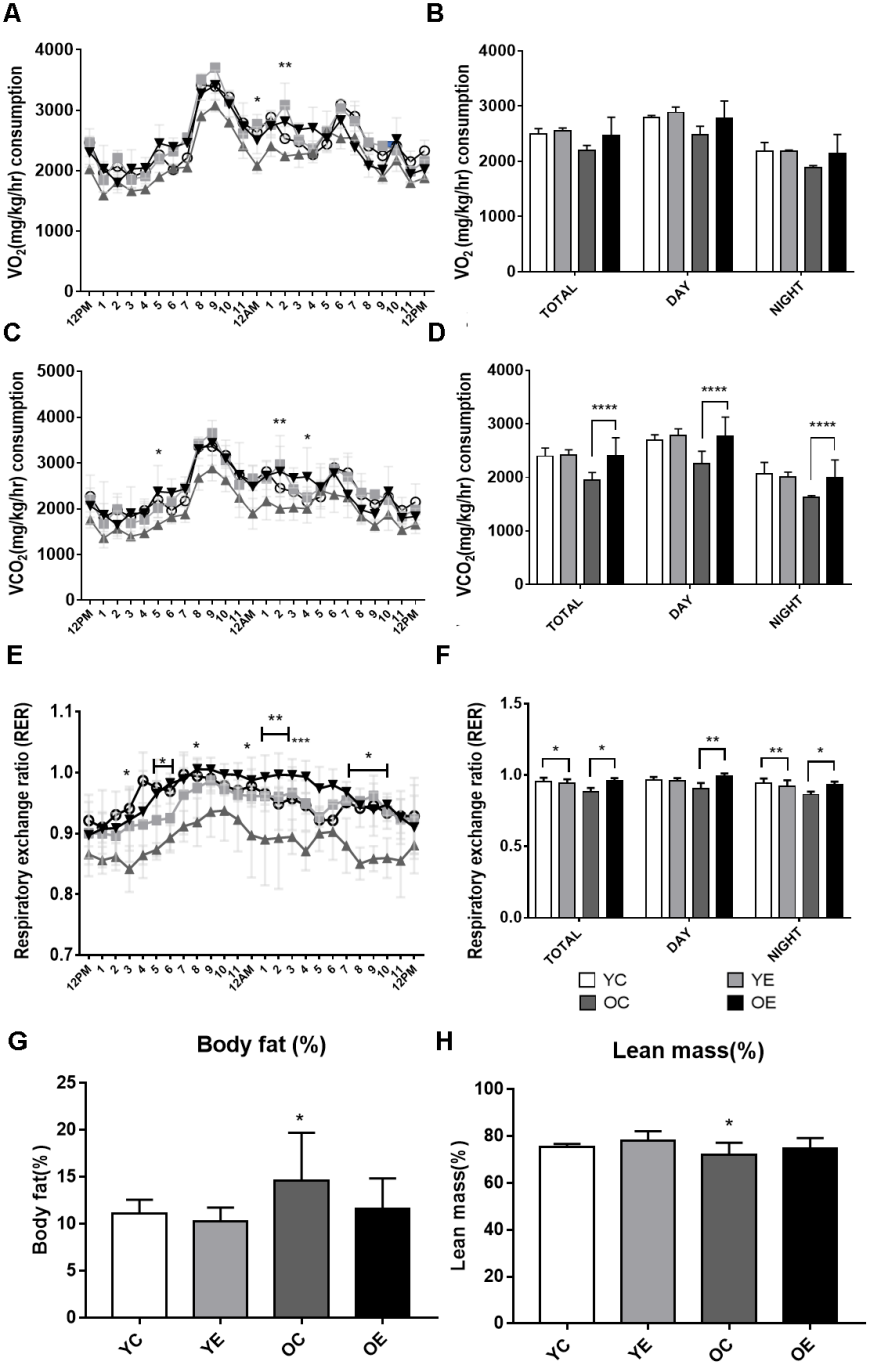
**Analysis of energy metabolism during exercise in young and aged mice.** (**A**–**F**) Metabolic measurements were performed in young control, young exercise, old control, and old exercise groups (*n* = 5, respectively) in CLAMS metabolic cages after 4-week of exercise. (**A**) Kinetic data for VO_2_ (mg/kg/hr) consumption are shown as mean for each time point in young control (YC; blue circles), young exercise (YE; red rectangles), old control (OC; green triangles) and old exercise (OE, purple reversed triangles) groups. (**B**) Average VO_2_ (mg/kg/hr) are shown for total, night (dark) and day (light) cycles. (**C**) Kinetic data for VCO_2_ (mg/kg/hr) production are shown as mean for each time point in YC, YE, OC, and OE groups. (**D**) Average VCO_2_ (mg/kg/hr) production are shown for total, night (dark) and day (light) cycles. (**E**) Kinetic data for respiratory exchange ratio (RER) is shown as mean for each time point in YC, YE, OC, and OE groups. (**F**) Average RER are shown for total, night (dark) and day (light) cycles. (**G**–**H**) Average percent body fat (**G**) and lean mass (**H**) for YC, YE, OC, and OE were measured using Minispec LF-50. *p < 0.05, **p < 0.01, ***p < 0.001, ****p < 0.0001.

To investigate the effect of exercise on the metabolic phenotypes of aged mice, 84-week-old male mice were subjected to one-hour treadmill running for 4 weeks (Figure 1). The average body weight was significantly higher (*p* < 0.05) in old mice than in young mice (Supplementary Figure 1A). During the 4-week experiment, body weight was decreased both in the old control (OC) and old exercise (OE) groups (*p* < 0.001), whereas no changes were observed both in young exercise (YE) compared to young control (YC) groups (Supplementary Figure 1A). We assessed the differences in metabolic parameters among the YC, YE, OC, and OE groups using metabolic chambers. There was no difference in activity in the metabolic chambers among groups (*p* > 0.05; Supplementary Figure 1B). Although the OC group showed reduced VO_2_ consumption at some time points (day 2, 12–1 a.m., *p* < 0.05; 2–3 a.m., *p* < 0.01; Figure 2A), there was no difference in the average VO_2_ consumption among the groups (*p* > 0.05; Figure 2B). Kinetic data for CO_2_ production showed differences at some time points (day 1, 4–5 p.m., *p* < 0.05; day 2, 1–2 a.m., *p* < 0.01 and 3–4 p.m., *p* < 0.05) between the OC and OE (Figure 2C). Average CO_2_ production was significantly reduced in the OC group compared to the YC, YE, and OE groups (*p* < 0.0001), regardless of the light/dark cycle (Figure 2D). The respiratory exchange ratio (RER), which was reduced in the OC group compared to the YC groups, was restored in the OE group (*p* < 0.05; Figure 2E, 2F). The fat mass percentage was increased in the OC group compared to the YC and YE groups (*p* < 0.05; Figure 2G) but was not significantly different in the OE compared to the YE and YC groups (*p* > 0.05; Figure 2G). The percentage of lean mass was reduced in the OC group compared to the YE group, while there was no change in the OE group compared to the YC and YE groups (*p* > 0.05; Figure 2H). These data imply that 4 weeks of exercise in old mice was sufficient to modify metabolic parameters similar to young mice.

### Exercise-induced changes in immune cells in the SVF derived from the adipose tissue of aged mice

**Figure 3:**
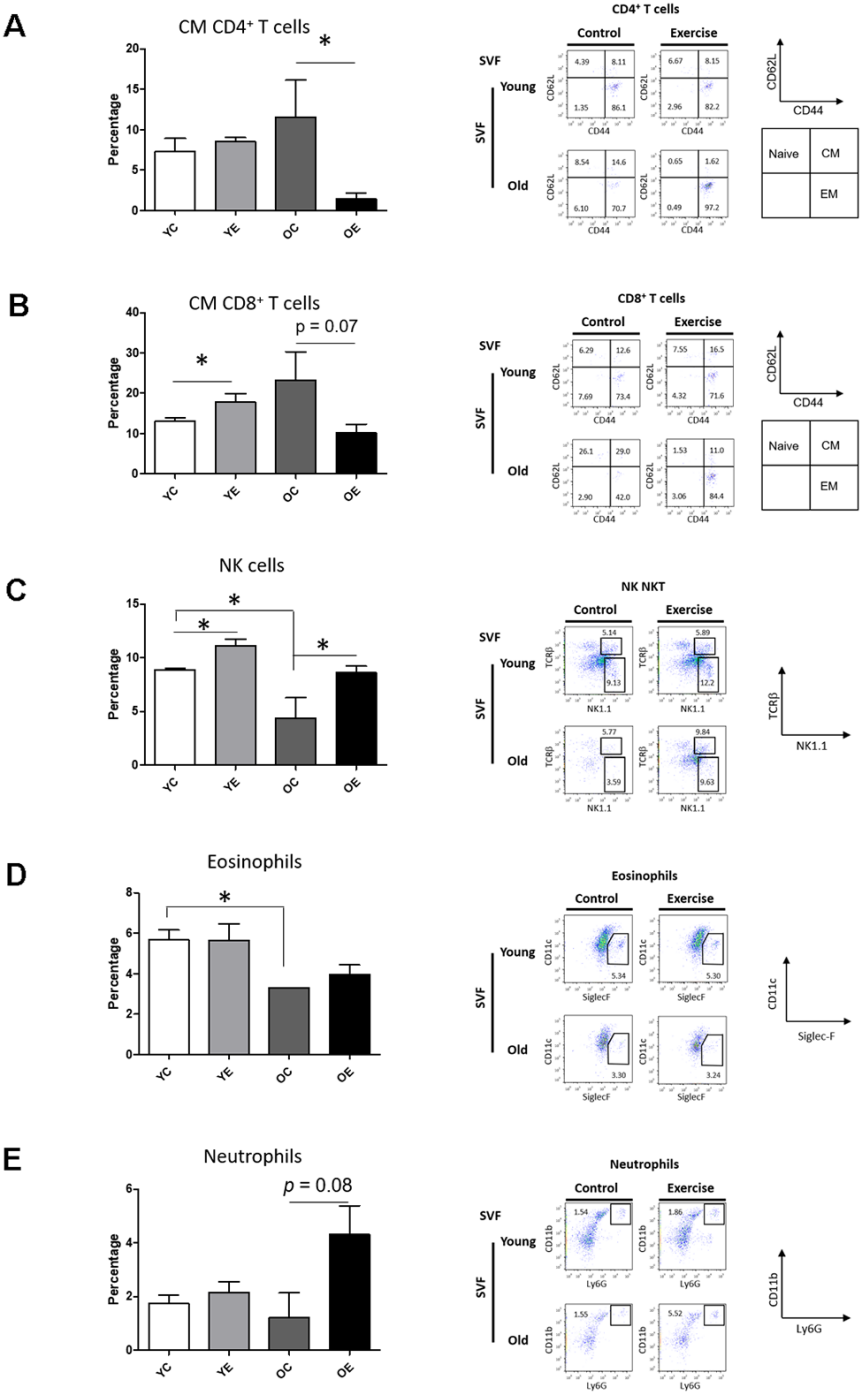
**Effect of exercise on Immune cell profiling in SVF and spleen of young and aged mice.** Bar graph and dot plots depict the frequencies of central memory (CM, CD62L^+^CD44^+^) CD4^+^ T cells (**A**); the frequencies of CM CD8^+^ T cells (**B**); the frequencies of NK (NK1.1^+^TCRβ^-^) cells (**C**); the frequencies of eosinophils (F4/80^+^Siglec-F^+^) (**D**); and the frequencies of neutrophils (F4/80^-^CD11c^-^CD11b^+^Ly6G^+^) (**E**) in SVF.

The adaptation of immune cells is linked to senescence accompanied by decreased metabolic function, especially within the susceptible SVF derived from adipose tissue [34]. Thus, we investigated the effect of exercise on the frequency of immune-cell subsets in the SVF from subcutaneous WAT of young and aged mice by flow cytometric analysis. Two-way ANOVA showed a marginal effect of the aging × exercise interaction (F_(1,8)_ = 5.317, *p* = 0.05) on central memory (CM) CD4^+^ T cells. Exercise significantly reduced the frequency of CM CD4^+^ T cells (*p* < 0.05) in aged mice compared to control (Figure 3A). There was no difference among groups in the frequencies of CD25^+^FoxP3^+^ regulatory T (Treg) cells, naïve CD4^+^ T cells, or effector memory (EM) CD4^+^ T cells (*p* > 0.05; Supplementary Figure 2). There was a significant effect of the aging × exercise interaction on CM CD8^+^ T cells (F_(1,8)_ = 5.433, *p* < 0.05). There was a marginal increase in the frequency of CM CD8^+^ T cells in the YE group compared to the YC group (*p* < 0.05; Figure 3B) and a slight decrease in the frequency of CM CD8^+^ T cells in the OE group compared to OC group (*p* = 0.07; Figure 3B). However, there were no differences between groups in the frequencies of naïve CD8^+^ T cells, and EM CD8^+^ T cells (*p* > 0.05; Supplementary Figure 2). There was no difference among the groups in the frequency of natural killer T (NKT) cells, B cells, M1 and M2 macrophage (*p* > 0.05; Supplementary Figure 2). However, there was a significant change in the frequency of NK cells, specifically in exercise (F_(1,8)_ = 5.433, *p* < 0.05) and aging (F_(1,8)_ = 11.428, *p* < 0.001; Figure 3C). Although there was a significant decrease in NK cells in the OC group compared to YC group (*p* < 0.05; Figure 3C), exercise increased the frequency of NK cells in both young and aged mice compared to the sedentary control (*p* < 0.05, respectively; Figure 3C). The main effect of aging had on the number of eosinophils (F_(1,8)_ = 14.499, *p* = 0.005; Figure 3D), which was seen as a decrease in old mice. Exercise increases the frequency of eosinophils in aged mice compared to control (*p* < 0.05; Figure 3D). There was a significant main effect of exercise (F_(1,8)_ = 9.223, *p* = 0.047; Figure 3E) on neutrophils among the groups. Exercise marginally increased the proportion of neutrophils in aged mice compared to the sedentary control (*p* = 0.08; Figure 3E).

### Exercise restored the p16 level in the adipose tissue of aged mice

**Figure 4:**
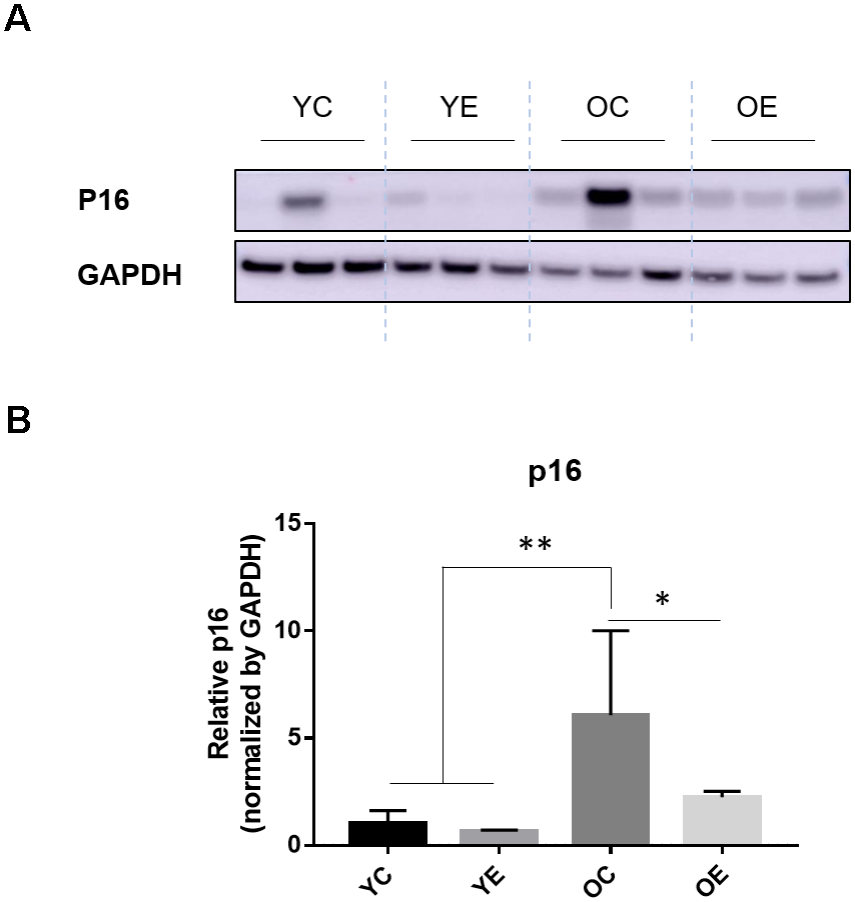
**Impact of exercise on a senescence marker, p16 and energy regulating enzyme in adipose tissues of aged mice.** (**A**) p16 and GAPDH were measured through immuno-blotting in the white adipose tissue (WAT) of both young and old mice after 4-week of treadmill exercise or sedentariness. Representative 3 samples per group are shown (total n=5, each group). (**B**) Bar graph depicts the mean (± standard error of the mean, SEM) intensity ratio of p16 to GAPDH bands measured using ImageJ program. *p < 0.05 compared to old sedentary controls, **p < 0.01 compared to young sedentary controls or exercise groups.

We analyzed the expression of p16, a cellular senescence marker, in adipose tissue after 4-week excise to determine that exercise affects aging. As expected, we found that p16 expression was increased in the OC group compared to the YC, YE groups (*p* < 0.001 for both), and the OE group (*p* < 0.05; Figure 4A, 4B).

### Effect of exercise on gene expression profiling of adipose tissue in aged mice

**Figure 5:**
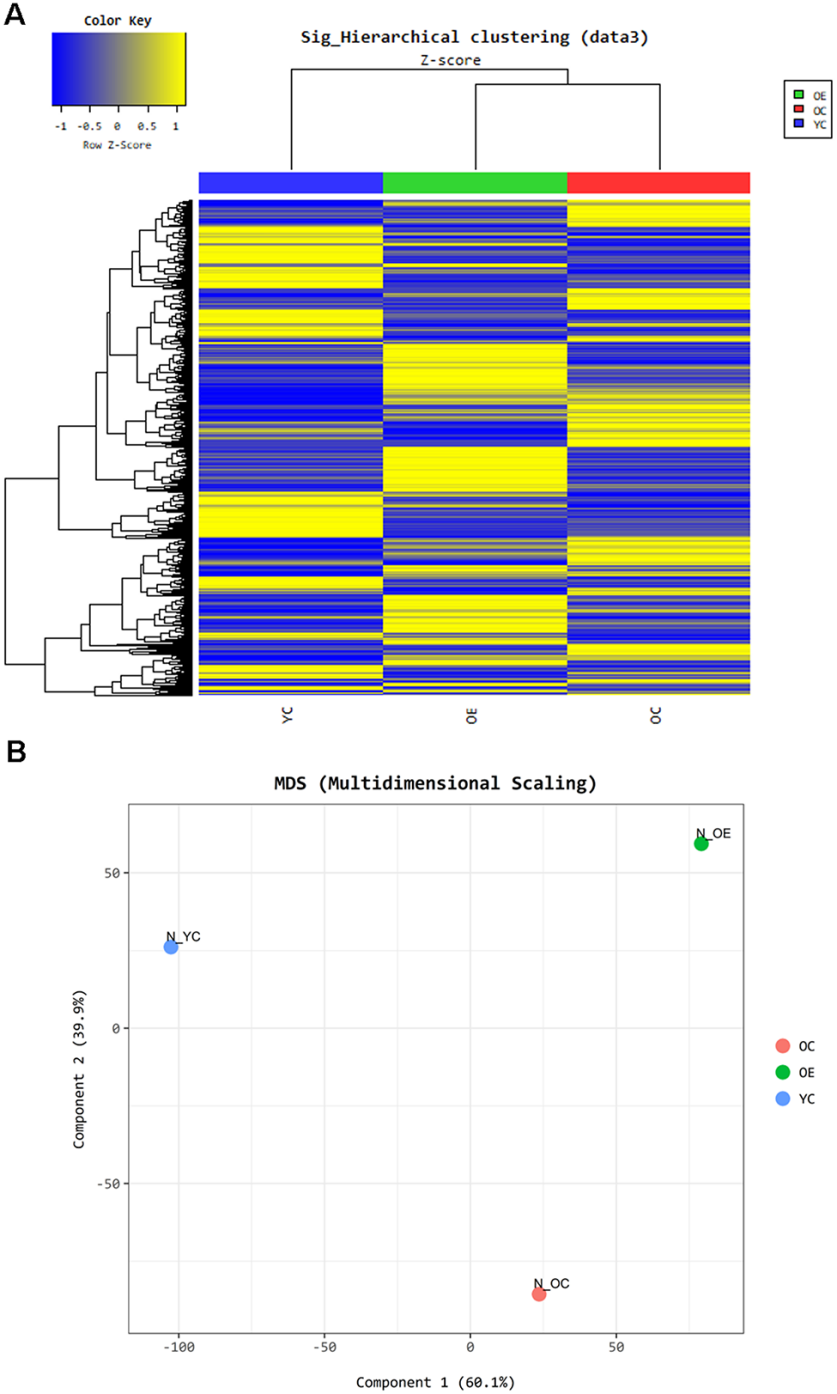
**Exercise-induced altered gene expression in adipose tissue of aged mice.** Gene ontology based- bioinformatic analysis (**A**) Heatmap represents grouping of genes through Hierarchical clustering (Euclidean Method, Complete Linkage) using the expression level (normalized value). (**B**) Panel shows the similarity between samples in two dimensions using the normalized signal of each sample.

**Figure 6:**
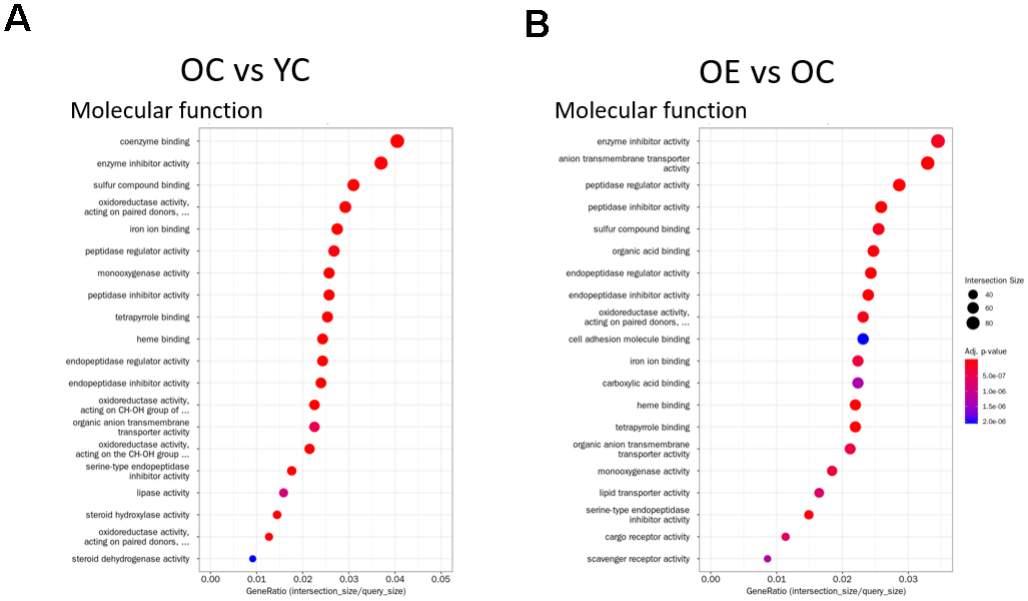
**Gene Ontology Enrichment analysis.** (**A**, **B**) Enrichment results for the top 20 GO terms in (**A**) OC vs YC and (**B**) OE vs OC that satisfy adjusted *p*-value < 0.05 for each GO category are plotted. Dot size indicates Gene Ratio.

To understand the effect of exercise on metabolic adaptation of adipose tissue, we analyzed transcriptome data from adipose tissue of aged mice after or without exercise. Using the fold change cut-off of ≥ 2, 1,506 and 1,334 genes were found to be upregulated and downregulated, respectively, in the OE group compared to the OC group, and 1,606 and 1,548 genes were upregulated and downregulated, respectively, in the OC group compared to the YC group. Gene expression between these three groups was distinct, and differentially expressed genes (DEGs) in OE and YC groups showed similar patterns compared to OC groups (Figure 5A). We tried to extract biological information from the multidimensional scaling analysis of DEGs. Distribution of ontologically representative gene sets revealed distinguishable distances among the groups (Figure 5B). Gene ontology (GO) and Kyoto Encyclopedia of Genes and Genomes (KEGG) pathway analyses showed that cytokine–cytokine receptor interactions, chemokine signaling pathways, inflammatory mediator regulation of TRP channels, and phagosome-related pathways were affected by exercise in aged mice (Supplementary Table 2). GO-based (REVIGO) bioinformatic analysis also revealed cytokine production and immune-system processes as two major categories in the OE group compared to the OC group (Supplementary Figure 3). Additionally, 20 GO terms were enriched among the DEGs identified in both the OC versus YC and OE versus OC comparisons based on their molecular function (Figure 6A, 6B). Terms such as “enzyme inhibitor activity”, “peptidase regulator activity”, “heme binding”, “iron ion binding”, “oxidoreductase activity”, and “monooxygenase activity” were commonly enriched in both sets of DEGs. On the other hand, terms such as “scavenger receptor activity” were specifically identified in the OE versus OC comparison (Figure 6B) and “lipase activity” specifically in the OC versus YC comparison (Figure 6A).

Changes in the proportions of immune cells, especially NK cells in the SVF after exercise, led us to explore changes in NK cell-related gene sets in adipocytes. Among NK cell-mediated cytotoxicity-related genes, 12 (*Cd48*, *Fcer1g*, *Fcgr4*, *Tyrobp*, *Ifngr2*, *Ncr1*, *Rac2*, *Itgb2*, *Lcp2*, *Nfatc2*, *Pik3cg*, *Prkcb*, and *Vav1*) were upregulated and 4 (*Cd244*, *Casp3*, *Gzmb*, and *Pik3r3*) downregulated in the OE group compared to the OC group (Table 1).

**Table 1:** NK cell mediated cytotoxicity related genes among DEGs in old exercise (OE) group compared to old control (OC) group. List of (**A**) up-regulated genes and (**B**) down-regulated genes.

**A Up-regulated genes**	
**Gene name**	**Description**
*Cd48*	CD48 antigen (*Cd48*)
*Fcer1g*	Fc receptor, IgE, high affinity I, gamma polypeptide (*Fcer1g*)
*Fcgr4*	Fc receptor, IgG, low affinity IV (*Fcgr4*)
*Tyrobp*	TYRO protein tyrosine kinase binding protein (*Tyrobp*)
*Ifngr2*	interferon gamma receptor 2 (*Ifngr2*)
*Ncr1*	natural cytotoxicity triggering receptor 1 (*Ncr1*)
*Rac2*	RAS-related C3 botulinum substrate 2 (*Rac2*)
*Itgb2*	integrin beta 2 (*Itgb2*)
*Lcp2*	lymphocyte cytosolic protein 2 (*Lcp2*)
*Nfatc2*	nuclear factor of activated T cells, cytoplasmic, calcineurin dependent 2 (*Nfatc2*)
*Pik3cg*	phosphoinositide-3-kinase, catalytic, gamma polypeptide (*Pik3cg*)
*Prkcb*	protein kinase C, beta (*Prkcb*)
*Vav1*	vav 1 oncogene (*Vav1*)

**B Down-regulated genes**	
**Gene name**	**Description**
*Cd244*	CD244 natural killer cell receptor 2B4(*Cd244*)
*Casp3*	caspase 3(*Casp3*)
*Gzmb*	granzyme B(*Gzmb*)
*Pik3r3*	phosphatidylinositol 3 kinase, regulatory subunit, polypeptide 3 (*p55*) (*Pik3r3*)

## DISCUSSION

Optimization of the immune system is critical for maintaining health. Indeed, the decrease in immunity that occurs with aging, termed immunosenescence [14, 15], contributes to geriatric disorders such as dementia, Parkinson’s disease, arthritis, osteoporosis, heart disease, high cholesterol, and metabolic syndromes [17]. In particular, cellular senescence-induced inflammation has been linked to dysfunction of fat tissue, which causes an imbalance in metabolic homeostasis [17, 18]. Several studies have demonstrated that exercise has anti-aging effects and can ameliorate metabolic disease [9, 10, 35]. However, it remains unclear how exercise leads to metabolic benefits in aging. Here, we evaluated whether changes in immunosenescence induced by exercise were associated with metabolic abnormalities in aging.

### Validation of anti-aging effect of exercise

It has been known that the expression of p16, as an *in vivo* marker of senescence, mediates cellular senescence [28, 36]. It was not surprising that exercise prevented the progression of senescence in the WAT of aged mice [37]. Consistent with the previous study [28, 37], p16 levels in WAT were increased in sedentary aged mice compared to sedentary young mice, and were decreased in aged mice after exercise. At the same time, the RER of exercised mice returned to the level of young mice. The RER was calculated by O_2_ consumption and CO_2_ production and is an indirect indicator of aerobic fitness. Additionally, body fat mass was reversed by exercise in aged mice. As shown in Supplementary Table 2, comparing differentially expressed genes related to biological processes in WAT of young and old mice, large metabolic changes are accompanying during aging. We found that exercise may affect metabolism in different directions, as the expression patterns of genes related to fatty acid biosynthesis such as *acsl4* and *olah*, which were increased in the old mice, decreased after 4 weeks of exercise. Overall, we can conclude that 4 weeks of treadmill exercise had a profound effect on improving energy metabolism and body composition by reducing senescence in adipose tissue.

### Exercise-induced immune cell alterations in the SVF of aged mice

The immune system, broadly consisting of the innate and adaptive immune systems, is a host defense system that responds specifically to external pathogens [38]. The innate immune response is the first line of defense, involving NK cells and phagocytic cells such as eosinophils, neutrophils, and macrophages. The adaptive immune response, represented by B and T cells, is mediated by antigen-specific defense mechanisms and takes several days for the complete immune response to develop. Immune cells also recognize and dispose of abnormal cells in our body. Both the innate and adaptive immune systems are strongly associated with aging and exercise [39]. To determine if the exercise-induced metabolic improvements in aged mice are related to the immune system, we investigated immune cells in the SVF of aged mice following exercise.

The most noticeable immunological changes associated with aging are defective function of T and B cells [40]. T-cell precursors are derived from the bone marrow and undergo lineage determination to become CD4^+^ (helper) or CD8^+^ (cytotoxic) T cells in the thymus. Many other age-associated changes in T-cell numbers and phenotypes have been reported [41]. Although not all T-cell compartments are equally affected by age, overall T-cell numbers decline with age as thymic involution leads to a decreased output of cells [41]. The decrease in naïve T-cell output from the thymus, increase in memory T cells from multiple antigenic encounters and homeostatic proliferation, and increase in regulatory T cells are profound qualitative and quantitative changes that occur with age [22, 42]. Lower numbers of hematopoietic stem cells and reduced output of antigen-specific naïve T cells from the thymus result in decreased numbers and proportions of naïve CD4^+^ and CD8^+^ T cells and increased numbers and proportions of late-stage differentiated EM CD4^+^ and CD8^+^ T cells during aging. In the elderly, however, an active lifestyle appears only to limit the accumulation of late-stage differentiated T cells and not to substantially affect the proportion of naïve T cells [43]. In accordance with a previous study, we did not observe a significant effect of aging or exercise on naïve CD4^+^ T cells, CM CD4^+^ T cells, or CD25^+^FoxP3^+^ regulatory T cells. However, we observed a marginal reduction in EM CD4^+^ T cells in the SVF of aged mice, indicating maladaptive immunity during senescence. Considering aged memory CD4^+^ T cells maintain a more catabolic state in lipid metabolism [44], these results provide evidence of disruption of fat metabolism in aged mice. Our data showed that exercise substantially increased CM CD8^+^ T cells only in the SVF of young mice; only a slight decrease was observed in older mice. The SVF of WAT is a reservoir for memory T cells, which protect against reinfection [45]. These findings support the idea that immunological memory, a critical step in the adaptive immune system, is boosted by exercise in young mice. Adipocytes directly interact with NKT cells by presenting lipid antigens and stimulate NK T cells to alleviate inflammation [46]. Dysfunction of NKT cells has been shown to lead to the development of autoimmune diseases such as diabetes, atherosclerosis, and cancer. However, in the present study, exercise and aging did not affect the proportion of NKT cells.

B cells play a critical role in immunosenescence via antibody production [47]. Although previous studies have demonstrated that B cells in the blood are notably impacted by exercise [48], the frequency was stable in the SVF of both young and aged mice after exercise or no exercise, suggesting that B cells might play a role in immune homeostasis in the SVF of WAT.

Low-grade inflammation in adipose tissue is closely associated with obesity and insulin resistance [49]. M1 macrophage inhibits proliferation of adipocytes and causes tissue damage, whereas M2 macrophage promotes proliferation of adipocytes and tissue repair [50]. A recent study suggested exercise-mediated switching of M1 to M2 macrophages reduced inflammation in obese mice [50], while other studies suggested that improvements of inflammation may involve an attenuation of both M1 and M2 macrophages in adipose tissue [51, 52]. Although we did not observe any differences in the proportion of M1 or M2 macrophages in the aged or exercised groups, experimental conditions such as duration and intensity of exercise, timing of dissection, sample type (SVF, WAT, and blood), and senescence in adipose tissue may have affected our results.

Neutrophils are abundant myeloid cells in the blood. Neutrophils, which serve as a link between phagocytosis and immunomodulatory responses, are increased by catecholamines during exercise [53]. Studies have shown that the number of neutrophils is not altered in healthy elderly [54, 55]. Consistent with previous studies, we observed that aging did not influence the number of neutrophils, while exercise marginally increased the number of neutrophils in aged mice.

The effects of aging and exercise on eosinophils, another circulating myeloid cell, are still unclear [56, 57]. In this study, exercise increased the proportion of eosinophils in aged mice. Because eosinophils play a critical role in the regulation of fat homeostasis and systemic metabolism, the exercise-mediated increase in eosinophils may affect the metabolism of WAT in aged mice.

Our finding that was most consistent with previous studies was the change in the proportion of NK cells. NK cells not only play a major role in virally infected cells but also eliminate abnormal cells, such as cancer and senescent cells. In the line with previous studies [16, 58], we observed a decreased proportion of NK cells in the SVF of aged mice. By contrast, an exercise-induced increase in NK cells was observed in young and aged mice. Because metabolic disorders prevent the ability of NK cells to regulate the local immune system [59], NK cells in the SVF of aged mice may be decreased because of fat accumulation in adipose tissue during aging. Jahn and colleagues [60] showed that weight loss in obese individuals via diet and exercise resulted in increased interferon-γ production by NK cells. Barra and colleagues [61] showed that high-intensity interval training increased the proportion and function of NK cells in obese women and mice. NK cells are also systemic innate immune cells defensing against viral infection as well as tumor surveillance [62]. In preparation for the recent corona virus (COVID-19) pandemic, the exercise to boost NK cells of vulnerable groups should be emphasized. Collectively, these studies suggest a critical role of exercise in restoring immune-cell profiles, especially that of NK cells, in the SVF of WAT, as well as a therapeutic potential of exercise in metabolic rejuvenation in aging.

### Exercise triggered an immune response in the adipose tissue of aged mice

The immune system works with other tissues to defend the body. Many studies have reported that exercise increases the number of NK cells with various anti-aging phenotypes [63]; however, little is known about the underlying physiological and genetic changes. To understand the effect of exercise-induced immune system changes in the SVF of adipose tissue and the improvement of energy metabolism in aged mice, we examined gene expression patterns in adipose tissue using microarray analysis. As expected, a high prevalence of functional annotation clusters, with greater enrichment scores, associated with cell projection assembly, wound healing, anion transport, cytokine production, and immune system process in the exercise group of aged mice. It should be noted that the expression levels of exercise-, immune-, and scavenging receptor-related genes were altered in the adipose tissue of aged mice. It has been suggested that exercise can affect the removal of aging-related problematic cells by regulating immune cells. KEGG pathway analysis confirmed the changes in various genes related to the function of NK cells. We confirmed an increase in genes related to NK cell function, such as *Cd48*, *Ncr1*, and *Fcer1g*; we also found that the expression of NK-cell-induced phagocytosis-related genes such as *Gzmb* and *Cd244* was decreased in adipose tissue by exercise. These complex changes may be due to dynamic changes such as proliferation, differentiation, and apoptosis in aged adipose tissue according to environmental metabolic requirements [64].

Recent studies have supported the role of immune cells in the removal of dead, senescent, or damaged cells [11, 14]. Although there is still no direct evidence that the changes in immune cells triggered by exercise provide metabolic benefits via the removal of senescent adipocytes, the increases in NK cells and neutrophils as well as the changes in NK cell-mediated cytotoxicity-related genes in adipose tissue suggest that immune cells can mediate local and systemic metabolic rejuvenation via exercise.

## MATERIALS AND METHODS

### Animals

The 9-week-old C57BL/6 (B6) young mice (control, YC, *n* = 9; exercise, YE, *n* = 8) and 84-week-old mice (control, OC, *n* = 8; exercise, OE, *n* = 8) were individually housed in standard conditions with food and water *ad libitum* as previously described [65]. Three mice of each group were not conducted for behavioral tests and they were only used for flow cytometry analysis. At the end of the scheduled experiments, each organ was dissected and sacrificed after deep anesthesia with isoflurane (Henry Schein Animal Health) with O_2_. Some organs were used for the brain study [65]. Epididymal adipose tissue was extracted for flow cytometric analysis. The experimental procedure was approved by the animal ethical review board of Seoul National University (SNU-171226-3).

### Exercise protocol

Before 4 weeks of treadmill exercise were performed, adaptation exercise was previously described [65], mice were familiarized with the treadmill for 15 min/session at a 0 m/min for 3 min, 5 m/min for 2 min and 8 m/min for 10 min and 6° incline once a day for 3 days prior to the experimental day. In 4 weeks of treadmill exercise, mice allocated to perform treadmill running were subject to 6° incline and for warm up, 2 min speed of 0 m/min, speed of 5 m/min, 8 m/min, 10 m/min for 1 min each. Then the mice ran 12 m/min for 30 min at first week, 2 m/min were increased every week, and cool down at 5 m/min for 2 min for 1 session (37 min). 2 session/day were performed. Between the session, there were at least 1 hr break time was given according to the previous studies [66, 67].

### Isolation of stromal vascular fraction from epididymal adipose tissue

Preparation of the SVF from WAT was performed as previously described [68]. In brief, WAT was minced with Iris scissors and digested with 2 mg/mL Collagenase I (Sigma-Aldrich) in DMEM (Sigma-Aldrich) at 37° C for 1 hr. Dulbecco’s Modified Eagle’s Medium (DMEM, Gibco) with 10% fetal bovine serum (FBS) was added to double the volume, floating adipocytes were discarded, and the remaining was filtered through a 100-μm mesh. The filtrate was centrifuged at 800 × *g* for 5 min, and the SVF pellet was resuspended in DMEM containing 10% FBS. SVF pellets were kept in the ice box.

### Flow cytometric analysis

SVF was first blocked on ice with staining buffer (phosphate-buffer saline, 0.5% bovine serum albumin) supplemented with 10% rabbit serum and pretreated with anti-CD16/CD32 (2.4G2) antibodies (Abs) to inhibit Fc receptor (FcR)-mediated Abs binding and then stained with various Abs to analyze the frequency of immune-cell subsets. The following anti-mouse Abs were used: Brilliant Ultraviolet (BUV) 395–anti-CD45, Brilliant Violet (BV) 711–anti-F4/80, fluorescein isothiocyanate (FITC)–anti-TCRβ, BV605–anti-CD11b, BUV737–anti-CD11c, BV421–anti-Siglec-F, Phycoerythrin (PE)–anti-Ly6G, BUV496–anti-B220, BUV805–anti-CD8, BUV496–anti-CD4, BV5605–anti-CD62L, BV421–anti-CD44, allophycocyanin (APC)–anti-CD25, BV711–anti-F4/80, BUV737–anti-CD3, FITC–anti-TCRβ, PE–anti-TCRγ, APC–anti-CD206, and PE-indodicarbocyanine–anti-NK1.1 (Supplementary Table 1 all reagents were from BD Biosciences except for the latter two, which were from eBioscience and BioLegend). Additionally, for staining FoxP3, cells were fixed with Fix/Perm buffer (eBioscience) and then permeabilized by Perm/Wash buffer (eBioscience) after staining appropriate surface antigens. Then cells were stained with PE-indotricarbocyanine–anti-FoxP3 (eBioscience). The Fixable Viability Dye eFluor 506 (eBioscience) was used to distinguish live versus dead cells prior to fixable and permeabilization. Stained cells were analyzed on a BD™ LSR Fortessa (BD Biosciences) instrument with FACSDiva™ software, and data were analyzed using FlowJo® software (TreeStar).

### Microarray and bioinformatic analysis

Each WAT from young sedentary (n = 4), old sedentary (n = 4), and old exercise (n = 4) groups were pooled and RNA purity and integrity were confirmed by ND-1000 Spectrophotometer (Nano-Drop, Wilmington, USA). The Affymetrix Whole transcript Expression array process was performed according to the protocol of the manufacturer (Gene Chip Whole Transcript PLUS reagent Kit). cDNA was synthesized according to the manufacturer’s method using Gene Chip Whole Transcript (WT) Amplification kit, and the hybridized array was scanned using a GCS3000 Scanner (Affymetrix). Signal values were calculated using Affymetrix® Gene ChipTM Command Console software. Gene enrichment and functional annotation analysis were performed on genes showing significant expression level (≥ 2-fold) based on gene ontology and KEGG pathway analysis, respectively. Gene Ontology Enrichment analysis was performed using g: Profiler tool (https://biit.cs.ut.ee/gprofiler/). The gene search procedure used the functional annotation category of REVIGO, uploaded the gene list, and selected an identifier from the official gene symbol. Species and background were selected for Mus musculus, and microarray data of this study were verified and compared.

### Energy balance

The mice were acclimatized to the cages for 2 days with freely access to water and food. Then oxygen consumption (VO_2_), carbon dioxide production (VCO_2_), respiratory exchange ratios (RER), and locomotor activity were measured using an indirect calorimetry system PHENOMASTER (TSE System). Mice in each chamber were maintained at a constant environmental temperature of 22° C.

### Body composition

Body fat and lean body masses were assessed by ^1^H magnetic resonance spectroscopy (MRS) using Minispec LF-50 from Bruker BioSpin (Billerica) at 24-hour after the last bout of exercise.

### Statistical analysis

Two-way ANOVA was used to detect significant main differences of aging and exercise. Considering the biological significant, paired groups for age and exercise were further analyzed by Student's *t*-test. Unpaired student's *t*-test was used for two-group comparisons.

## Supplementary Material

Supplementary Figures

Supplementary Table 1

Supplementary Table 2
