# Supplementary material for: Exercise reduces metabolic burden while altering the immune system in aged mice
Source: Aging (Albany NY). 2021 Jan 6;13(1):1294–313. doi: 10.18632/aging.202312 (PMC7834985; doi:10.18632/aging.202312)
Supplement: Supplementary Figures [file aging-13-202312-s001.pdf]

SUPPLEMENTARY FIGURES

A

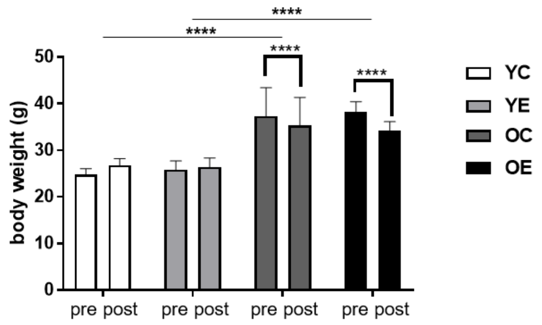

B

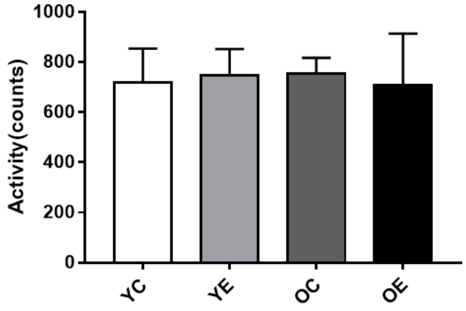

Supplementary Figure 1. (**A**) Body composition change with or without 4-week of treadmill training. (**B**) The average (mean ± SD) x, y, and z activity. ****$p < 0.0001$.

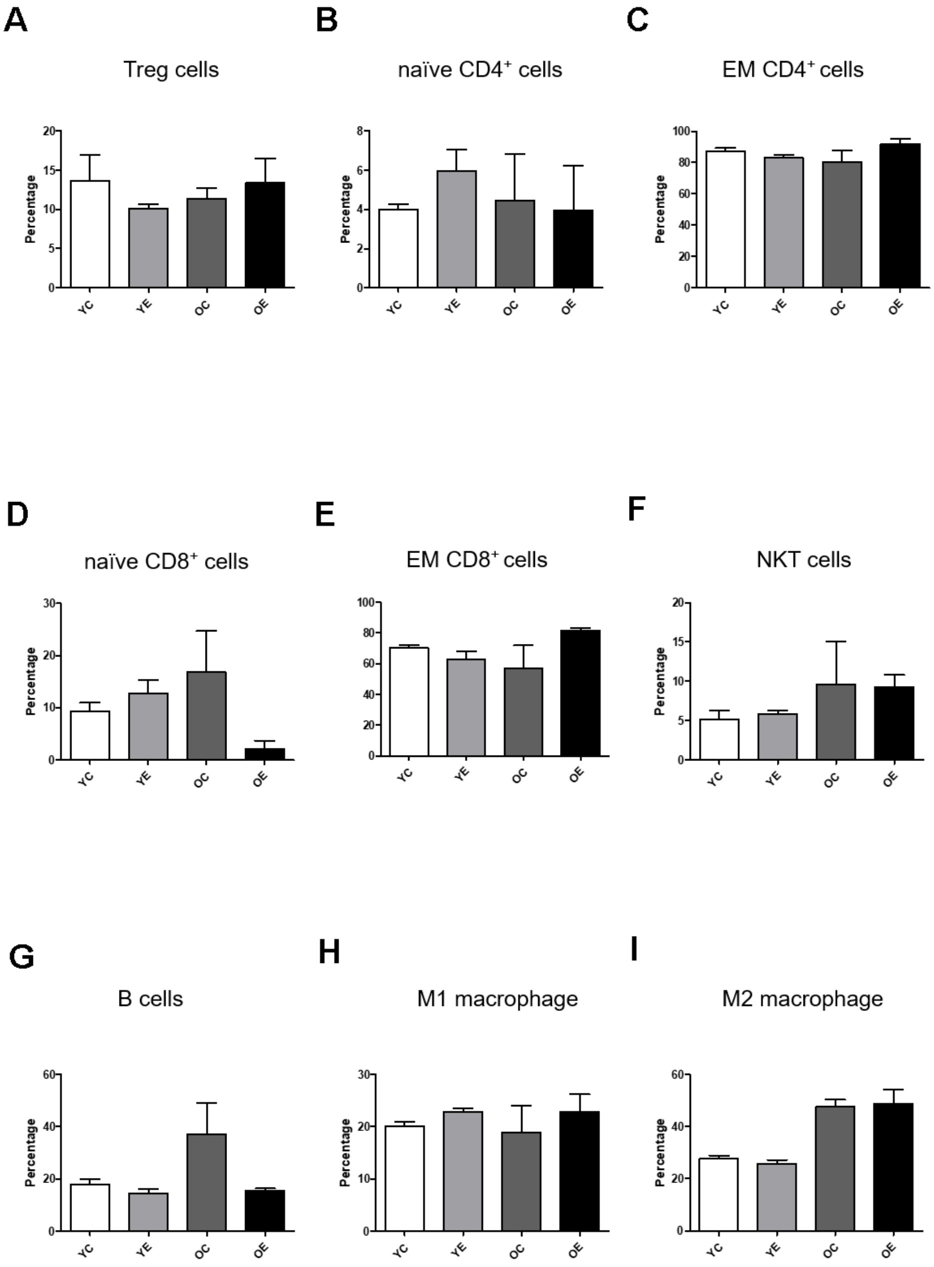

Supplementary Figure 2. Effect of exercise on immune cell profiling in SVF of young and aged mice. Bar graph depict the mean frequencies (± SEM) of (**A**) Treg, (**B**) naïve $CD4^+$ cells, (**C**) EM $CD4^+$ cells, (**D**) naïve $CD8^+$ cells, (**E**) EM $CD8^+$ cells, (**F**) NKT cells, (**G**) B cells, (**H**) M1 macrophage, (**I**) M2 macrophage in SVF.

A

OC vs YC

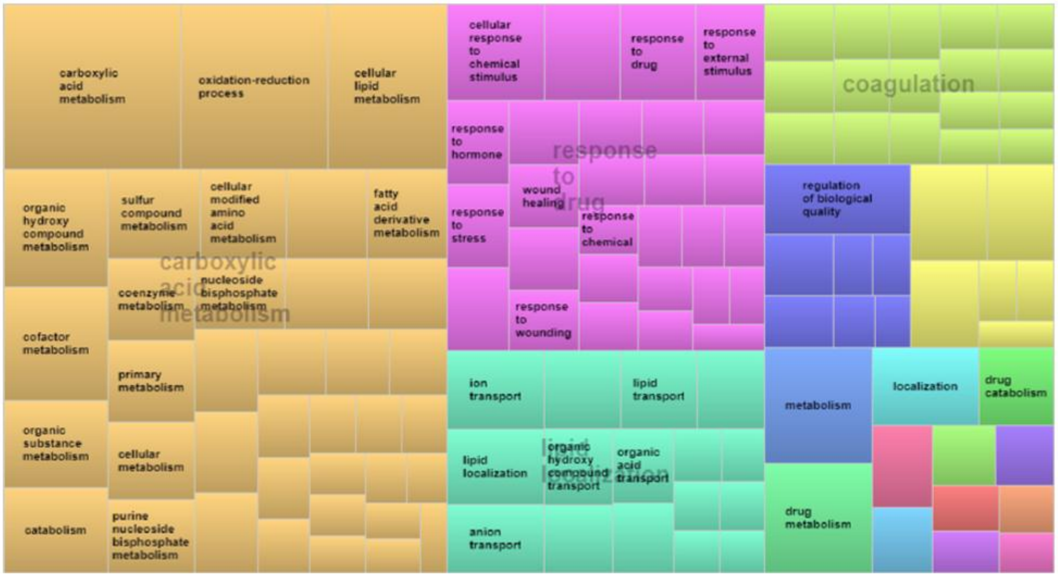

B OE vs OC

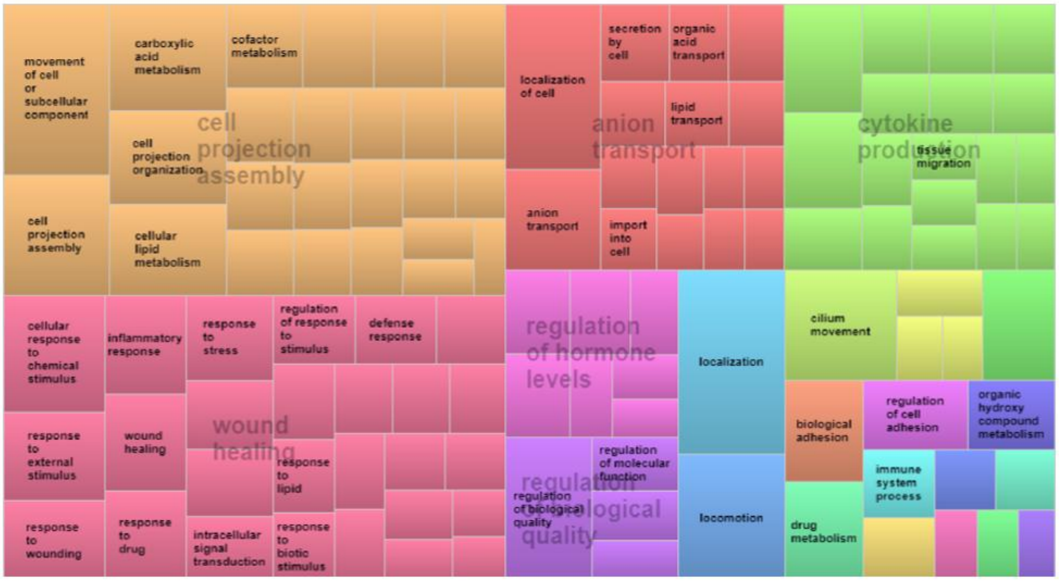

Supplementary Figure 3. Gene ontology based- treeMap view of REVIGO. Exercise-induced altered gene expression in adipose tissue of aged mice. (**A**) OC versus YC (**B**) OE versus OC. Size of the rectangles may be adjusted to reflect the frequency of the gene ontology term in the underlying Gene Ontology Annotation Database.
