# Supplementary material for: Exercise reduces metabolic burden while altering the immune system in aged mice
Source: Aging (Albany NY). 2021 Jan 6;13(1):1294–313. doi: 10.18632/aging.202312 (PMC7834985; doi:10.18632/aging.202312)
Supplement: Supplementary Table 1 [file aging-13-202312-s002.pdf]

## SUPPLEMENTARY TABLE

**Supplementary Table 1. Antibodies used to identify the given immune cell type molecularly.**

| Immune Cell | Type Molecular Identification Scheme |
|---|---|
| TCRgd | CD45.2+ F4/80- CD3+ TCRβ– TCRγ+ |
| CD8+ | CD45.2+ F4/80- CD3+ TCRβ+ CD4- CD8+ |
| Treg CD4+ | CD45.2+ CD4+ CD25+ Foxp3+ |
| Naive CD4+ | CD45.2+ CD4+ CD25- Foxp3- CD62Lhi CD44lo |
| Activated CD4+ | CD45.2+ CD4+ CD25- Foxp3- CD62L lo CD44hi |
| NKT | CD45.2+ NK1.1+ TCRβ+ |
| NK | CD45.2+ NK1.1+ TCRβ- |
| B cells | CD45.2+ NK1.1- CD19+ |
| Eosinophil | CD45.2+ F4/80+ Siglec-F+ |
| Neutrophil | CD45.2+ F4/80- CD11c- CD11b+ Ly6G+ |
| M2 ATM | CD45 .2+ F4/80+ CD11cmed CD206+ |
| M1 ATM | CD45.2+ F4/80+ CD11chi CD206- |
| DN (Double-negative) ATM | CD45.2+ F4/80+ CD11c- CD206 |
