# Supplementary material for: Exercise reduces metabolic burden while altering the immune system in aged mice
Source: Aging (Albany NY). 2021 Jan 6;13(1):1294–313. doi: 10.18632/aging.202312 (PMC7834985; doi:10.18632/aging.202312)
Supplement: Supplementary Table 2 [file aging-13-202312-s003.docx]

**Supplementary Table 2. Exercise-induced altered genes expression in adipose tissue of aged mice by KEGG pathway (a) old control (OC) group compared to young control (YC) group, (b) old exercise (OE) group compared to old control (OC) group.**

a. OC vs YC

| Map ID | Map Name | Number of Genes | P-Value | Bonferroni | FDR |
| --- | --- | --- | --- | --- | --- |
| 01100 | Metabolic pathways | 490 | 4.9731E-205 | 1.6361E-202 | 1.6361E-202 |
| 05200 | Pathways in cancer | 108 | 1.54219E-26 | 5.07379E-24 | 1.26845E-24 |
| 04151 | PI3K-Akt signaling pathway | 74 | 3.49695E-19 | 1.1505E-16 | 9.58748E-18 |
| 05165 | Human papillomavirus infection | 68 | 9.10075E-16 | 2.99415E-13 | 1.36098E-14 |
| 04714 | Thermogenesis | 57 | 8.77217E-18 | 2.88605E-15 | 1.92403E-16 |
| 04610 | Complement and coagulation cascades | 51 | 3.89383E-28 | 1.28107E-25 | 6.40536E-26 |
| 05204 | Chemical carcinogenesis | 51 | 8.22146E-28 | 2.70486E-25 | 9.01621E-26 |
| 00140 | Steroid hormone biosynthesis | 48 | 2.85219E-26 | 9.38371E-24 | 1.87674E-24 |
| 05205 | Proteoglycans in cancer | 48 | 2.59372E-14 | 8.53333E-12 | 3.04762E-13 |
| 04510 | Focal adhesion | 47 | 3.78333E-14 | 1.24472E-11 | 4.29212E-13 |
| 04015 | Rap1 signaling pathway | 46 | 1.2425E-12 | 4.08781E-10 | 1.2023E-11 |
| 04010 | MAPK signaling pathway | 46 | 1.0433E-08 | 3.43244E-06 | 5.36319E-08 |
| 03320 | PPAR signaling pathway | 45 | 4.23249E-24 | 1.39249E-21 | 2.32082E-22 |
| 04024 | cAMP signaling pathway | 45 | 4.85599E-12 | 1.59762E-09 | 4.3179E-11 |
| 00830 | Retinol metabolism | 44 | 1.09334E-22 | 3.5971E-20 | 5.13871E-21 |
| 05168 | Herpes simplex virus 1 infection | 44 | 0.000599546 | 0.197250716 | 0.001038162 |
| 01200 | Carbon metabolism | 43 | 2.61751E-18 | 8.61162E-16 | 6.15116E-17 |
| 04932 | Non-alcoholic fatty liver disease (NAFLD) | 43 | 1.9129E-15 | 6.29343E-13 | 2.62226E-14 |
| 05010 | Alzheimer disease | 43 | 1.19561E-13 | 3.93356E-11 | 1.31119E-12 |
| 00983 | Drug metabolism - other enzymes | 42 | 1.61159E-21 | 5.30213E-19 | 6.20218E-20 |
| 04750 | Inflammatory mediator regulation of TRP channels | 42 | 6.88076E-17 | 2.26377E-14 | 1.33163E-15 |

b. OE vs OC

| Map ID | Map Name | Number of Genes | P-Value | Bonferroni | FDR |
| --- | --- | --- | --- | --- | --- |
| 01100 | Metabolic pathways | 339 | 1.1419E-119 | 3.7226E-117 | 3.7226E-117 |
| 05200 | Pathways in cancer | 96 | 8.34111E-26 | 2.7192E-23 | 9.06401E-24 |
| 04151 | PI3K-Akt signaling pathway | 63 | 4.71447E-17 | 1.53692E-14 | 2.56153E-15 |
| 05165 | Human papillomavirus infection | 54 | 3.94192E-12 | 1.28507E-09 | 5.84122E-11 |
| 04080 | Neuroactive ligand-receptor interaction | 51 | 3.83925E-11 | 1.2516E-08 | 3.86353E-10 |
| 04060 | Cytokine-cytokine receptor interaction | 48 | 5.95883E-12 | 1.94258E-09 | 8.44599E-11 |
| 04145 | Phagosome | 47 | 2.47195E-18 | 8.05857E-16 | 1.61171E-16 |
| 04010 | MAPK signaling pathway | 47 | 1.56073E-11 | 5.08799E-09 | 1.96573E-10 |
| 04610 | Complement and coagulation cascades | 46 | 4.58575E-27 | 1.49496E-24 | 7.47478E-25 |
| 04510 | Focal adhesion | 45 | 1.28945E-15 | 4.2036E-13 | 6.00514E-14 |
| 04144 | Endocytosis | 44 | 3.91094E-11 | 1.27496E-08 | 3.86353E-10 |
| 04142 | Lysosome | 43 | 1.33773E-20 | 4.36099E-18 | 1.09025E-18 |
| 05152 | Tuberculosis | 42 | 3.65927E-15 | 1.19292E-12 | 1.49115E-13 |
| 04015 | Rap1 signaling pathway | 42 | 7.35311E-13 | 2.39711E-10 | 1.33173E-11 |
| 04024 | cAMP signaling pathway | 41 | 3.14092E-12 | 1.02394E-09 | 5.1197E-11 |
| 04062 | Chemokine signaling pathway | 40 | 1.26144E-12 | 4.11228E-10 | 2.16436E-11 |
| 04810 | Regulation of actin cytoskeleton | 40 | 1.30236E-11 | 4.24571E-09 | 1.76904E-10 |
| 04514 | Cell adhesion molecules (CAMs) | 38 | 4.16062E-13 | 1.35636E-10 | 9.14657E-12 |
| 04020 | Calcium signaling pathway | 37 | 3.13498E-11 | 1.022E-08 | 3.40668E-10 |
| 05163 | Human cytomegalovirus infection | 37 | 2.7085E-08 | 8.82972E-06 | 1.27967E-07 |
| 04750 | Inflammatory mediator regulation of TRP channels | 36 | 4.12767E-15 | 1.34562E-12 | 1.49513E-13 |
